# Insights About Interactions between Microplastics and Humic Acids: An Approach Theoretical Modeling and Experimental

**DOI:** 10.1021/acsomega.5c12106

**Published:** 2026-07-04

**Authors:** Guilherme de S. Parente, Leonardo P. da Silva, Lucas L. Bezerra, José Osmar de S. Júnior, Caroline B. Reinaldo, Christian P. Almeida, Norberto de Kassio V. Monteiro, Laercio L. Martins, Andre H. B. Oliveira

**Affiliations:** † Environmental Studies Laboratory (LEA), 28121 Federal University of Ceará (UFC), Fortaleza, Ceará 60440-900, Brazil; ‡ Theoretical Chemistry Group (GQT), 28121 Federal University of Ceará (UFC), Fortaleza, Ceará 60440-900, Brazil; § Laboratory of Petroleum Engineering and Exploration (LENEP), North Fluminense State University (UENF), Macaé, Rio de Janeiro 27932-125, Brazil

## Abstract

Microplastics (MPs) are ubiquitous and persistent pollutants that interact with various environmental components, especially with organic matter such as humic acid (HA) in sediments. This interaction is critical, for example, in estuarine sediments, which are key areas for MP deposition and where the presence of HA is frequently detected during extraction procedures. Considering the potential contact between HA and MPs, understanding their interaction mechanisms is essential. The present study employed both experimental and computational approaches to investigate the interaction between polyethylene (PE), polypropylene (PP), and polystyrene (PS) microplastics with HA. The experimental analyses, together with statistical evaluation, provided a more detailed view of interaction trends involving HA and MPs across the environmental parameters studied, particularly sediment granulometry (gravel, sand, and mud), organic carbon (OC), and organic matter (OM) revealing a clear correlation. To further investigate these interactions, computational analyses were employed. The computation also shows the clear tendency for HA-MP interactions, predominantly governed by van der Waals forces. Interaction potential energy (IPE) data suggest that PS exhibits a higher affinity with HA (−316 kJ mol^–1^) than PP (−254 kJ mol^–1^) or PE (−195 kJ mol^–1^). Additionally, hydrogen bonding data indicate a greater reduction in HA-water hydrogen bonds for PS, favoring HA-PS interactions. Visualizations of these interactions confirmed the presence of van der Waals interactions, as well as stronger interactions involving between HA and PS, primarily involving oxygen atoms in HA. These findings highlight the affinity between HA and MPs, particularly PS, and emphasize the need for further studies on the environmental risks posed in environmental matrices, such as estuarine environments.

## Graphical abstract

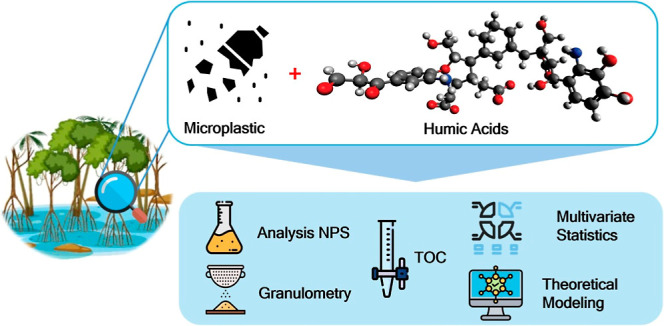

## 1 Introduction

Plastics are synthetic polymers widely used for their durability, low cost, and versatility. They are essential in sectors such as health, food, communications, and the automotive industry, among others.^1^ However, improper disposal and low biodegradability generate negative impacts on the environment, as well as aesthetic, economic, and human health damage, making plastic pollution one of the most significant global environmental challenges.^1–4^ Despite global efforts, plastic production continues to increase, with around 413 million tons of plastic in 2023,^5^ highlighting an urgent need for effective solutions.

Microplastics (MPs), plastic particles ranging from 1 μm to 5 mm, are persistent pollutants found in environments ranging from coastal and marine areas to rocky mountain ranges.^6,7^ They vary in size, color, and shape, including granules, fibers, fragments, and films.^8,9^ Their characterization includes optical microscopy for morphological analysis (color, shape, and texture) and Fourier transform infrared spectroscopy (FTIR) for chemical characterization of polymer types,^10^ such as polyethylene (PE), polyethylene terephthalate (PET), polystyrene (PS), and polypropylene (PP), among others.^9,11^ MPs attract attention due to their persistence, bioaccumulation, toxicity, and dispersion capacity in various matrices.^3,8,12^ These particles can be ingested, inhaled, or absorbed through the skin, resisting metabolization and accumulating through the food chain, causing significant damage.^9^ For example, in birds, MPs induce false sensations of satiety, suffocation, and endocrine disruption.^13,14^ In marine organisms, such as fish, they have been linked to neurotoxicity, growth retardation, and behavioral abnormalities.^14,15^ In humans, studies have shown that MPs can cause cytotoxicity, genotoxicity, oxidative stress, and chronic inflammation.^15–17^ These effects may contribute to endocrine dysfunction, as well as to gastrointestinal, hepatic, respiratory, and reproductive disorders, among others. Moreover, environmental contaminants sorbed on the surface of MPs can intensify their toxic effects, potentially increasing the risk of serious health outcomes.^17^ Studies have shown that MPs can act as vectors of contaminants, including polychlorinated biphenyls (PCBs), dichlorodiphenyltrichloroethane (DDT), polycyclic aromatic hydrocarbons (PAHs), heavy metals, antibiotics, and antidepressants.^18–20^ Furthermore, when introduced into the environment, MPs interact with the natural components present in environmental matrices, such as sediments, directly affecting their transport, distribution, and fate.^10,21^ This interaction is particularly critical in estuarine sediments, key areas for MP deposition, where their presence threatens the biodiversity characteristic of these ecosystems.^22,23^ These sediments are characterized by high levels of organic matter (OM) and fine sediments, act as geosorbents for hydrophobic organic compounds (HOCs), heavy metals, and MPs.^23–25^ Among the components involved in this process, there is the humic acid (HA), an organic fraction commonly found in estuarine sediments that presents aromatic rings and functional groups, such as hydroxyl, amine, and carboxyl.^26^

The presence of a variety of functional groups in HA provides abundant potential binding sites for organic pollutants, metals, and MPs.^27–31^ Due to this characteristic, HA can compete with various species in the environment. For example, Madeira et al.^29^ in their study evaluating the sorption capacity of fipronil (insecticide) on the surface of polyethylene (PE), observed that fipronil is readily sorbed via CH-pi in pure water. However, in the presence of HA, the sorption capacity was significantly reduced. This reduction was attributed to the interaction between MPs and HA, as evidenced by the complete coating of MP surfaces with HA, which blocks the available sorption sites for fipronil. Similarly, Chen et al.^12^ evaluated the behavior of a system containing HA, MPs, and calcium ions (Ca^2+^). Initially, Ca^2+^ complexes with the carboxyl groups present in HA. However, upon the addition of MPs, this coordination weakens due to steric hindrance.^12^ Therefore, the numerous binding sites of HA can alter the distribution and environmental availability of contaminants. These studies emphasize the crucial role of HA-MP interaction, highlighting the need for continued investigation into these mechanisms, which are still not fully understood. Particularly regarding HA-MP interactions, previous studies have demonstrated that the nature of these interactions varies depending on the type of polymer.^12,30,31^ For example, experimental studies suggest that polystyrene (PS) interacts with HA through pi–pi interaction, hydrogen bonding, and electrostatic interactions.^30,31^ With polyvinyl chloride (PVC) MPs, it interacts through electrostatic interactions, hydrogen bonding, and halogen bonding.^31^ The occurrence of these interactions is further supported by sediment extraction experiments, which confirm that HA is naturally present in the sediment matrix.^32,33^ As MPs also tend to accumulate in these environments, HA can play a decisive role in their environmental dynamics. Understanding the mechanism of HA-MP interaction is essential for elucidating the factors that stabilize these associations and clarifying their role in the distribution and persistence of MPs in the environment. One way to investigate the depositional processes of MPs is through theoretical approaches.

Computational techniques, such as molecular dynamics (MD), have gained prominence in studies involving MP interactions.^12,34^ This approach provides key insights that support the development of mitigation strategies for reducing the environmental impacts. Our MD studies have yielded novel insights into noncovalent interactions between MPs and HA, including the decomposition of interaction energies, the influence of water hydrogen bonds, and a topological analysis of electron density using IGM. These studies illuminate the mechanisms of adsorption and transport as they relate to each MP under investigation and provide key insights to support the development of mitigation strategies to reduce environmental impacts. Thus, this study aims to investigate the molecular dynamics and depositional processes of MPs in estuarine sediments by integrating theoretical and experimental methods. In doing so, it seeks to elucidate the interactions and their mechanisms, with the expectation that the results will contribute to the development of effective mitigation strategies, promoting the preservation of estuarine ecosystems.

Thus, this study combines environmental observations, multivariate statistical analyses, and molecular simulations to investigate whether the interaction trends observed in estuarine sediments can be mechanistically explained at the molecular level.

## 2 Materials and Methods

### 2.1 Study Area

The study was conducted in the Environmental Protection Area (APA) of the Pacoti River, located in the estuary of the Pacoti River, covering the municipalities of Fortaleza, Eusébio, and Aquiraz in the state of Ceará, in the northeast region of Brazil, with an area of 2916.97 ha. The APA of the Pacoti River was created to preserve the local ecosystems due to their fragile ecological balance, which requires special protection from both public authorities and society. This region is rich in biodiversity, featuring mangroves, dunes, tabuleiro forest, and riparian vegetation.^35^ Mangroves, in particular, play a crucial role in particle retention due to their richness in organic components such as HA, functioning as sinks for MPs.^36,37^ However, this region faces several environmental challenges resulting from human activities, such as condominium construction, improper waste disposal, and vehicle traffic over the dunes, all of which threaten the integrity of the local ecosystems.^35^ Given this scenario, the area is highly susceptible to the presence of MPs and HA, providing an opportunity to study their dynamics and inform the development of mitigation strategies and environmental management. The map of the study area is shown in Figure 1.

**Figure 1:**
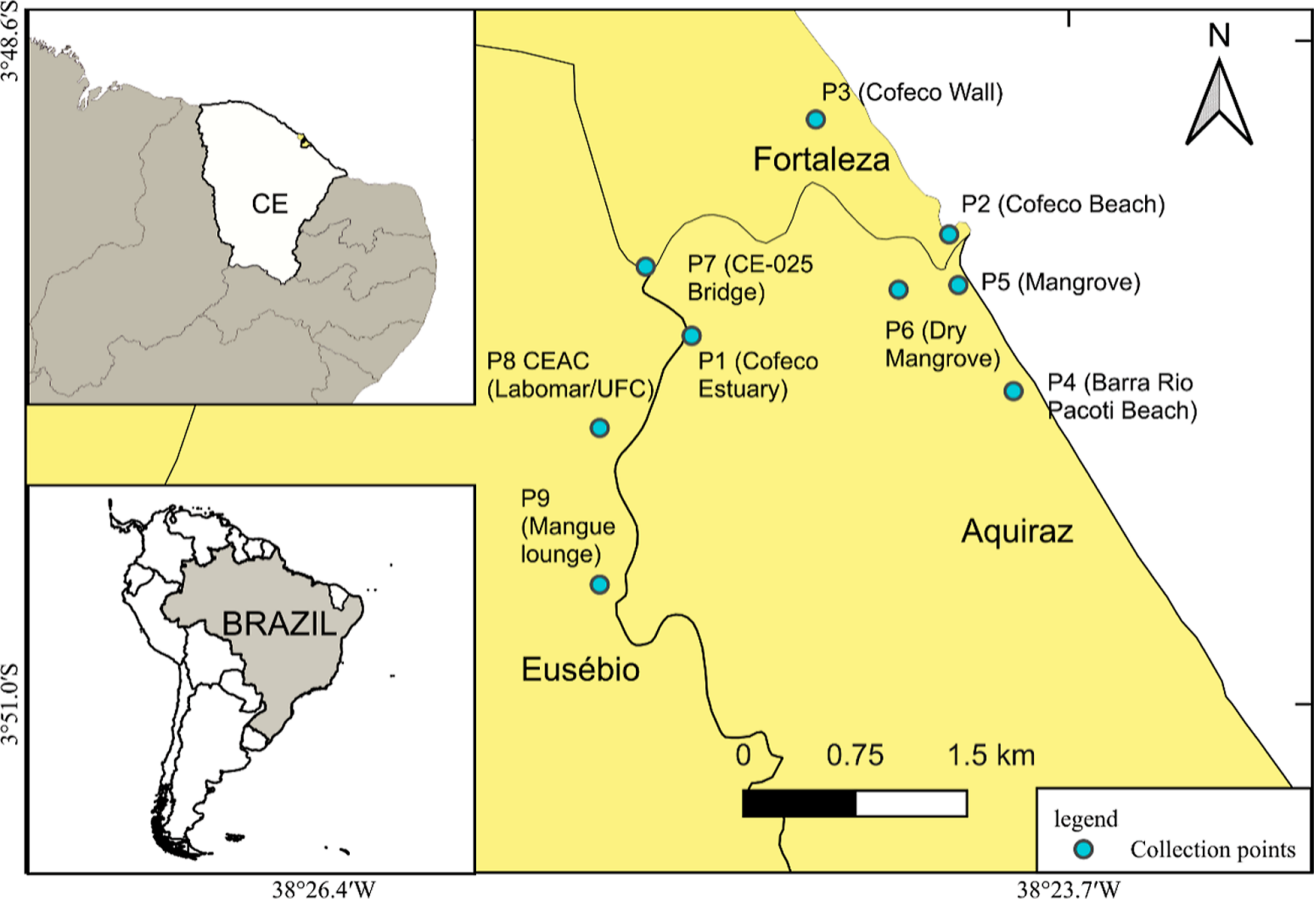
Sampling sites in Fortaleza, Eusébio and Aquiraz, State of Ceará, Northeastern Brazil. Map created using QGIS 4.0, an open-source geographic information system (GIS) software. Available at: https://www.qgis.org.

### 2.2 Sampling Sites

Nine collection points were chosen within the APA of the Rio Pacoti region (P1 to P9, Figure 1). This study area features diverse environmental characteristics, and the selected points were intended to represent the variety found within the APA. These points include areas near the estuary mouth, such as Cofeco Beach (P2), Cofeco Wall (P3), Barra Rio Pacoti Beach (P4), Mangrove (P5), and Dry Mangrove (P6), as well as surrounding ecosystems, Cofeco Estuary (P1), such as CE-025 Bridge (P7), Center for Coastal and Environmental Studies (CEAC) (Labomar-UFC) (P8), and Mangue Lounge (P9). The selection reflects the characteristics of estuarine regions, ranging from beaches, which have greater human access, to mangroves, which generally experience less human interference, enabling a detailed analysis of the APA. Furthermore, this sampling campaign (March 2023) represents a specific snapshot of this estuarine system, and therefore, the observed distributions of microplastics and humic acids may reflect the specific hydrological and environmental conditions prevailing during that period. The collection points and geographic coordinates are presented in Table S1.

### 2.3 Sampling Method

Sampling was conducted in March 2023 in the superficial sediment layer (5 cm depth), covering supralittoral areas exposed to the air. A 50 × 50 cm area was used to delimit the sampling area, within which the sediment was carefully removed with a stainless-steel spoon, avoiding any contact with plastic materials to minimize contamination risk. The quartering technique was applied to ensure sample representativeness and homogeneity. The collected material was stored in previously sterilized glass containers and later transported to the laboratory for further analysis.^37^

### 2.4 Sample Analysis

#### 2.4.1 Analysis of MPs

Samples were collected, taken to the laboratory, and subjected to evaporation in an oven at 100 °C until reaching a constant weight. The dried samples were then sieved through 4.75 mm meshes. The dry weights of the samples are presented in Table S2.

Subsequently, the samples were subjected to organic matter digestion using 20 mL of 30% hydrogen peroxide (H_2_O_2_) under heating at 75 °C on a hot plate, following the methodology of Masura et al.^38^ This procedure was repeated multiple times until the organic matter was completely digested. Subsequently, the sample was transferred to a glass beaker, where they were subjected to a concentrated sodium chloride (NaCl) solution with a density of 1.202 g cm^–3^ to separate MPs via density-based separation over 48 h. After flotation, the microplastics were separated and collected in Petri dishes for later visual analysis under a microscope. Qualitative and quantitative analysis of MPs was performed using optical microscopy with a Physics binocular stereo microscope, featuring a 40× magnification and equipped with a high-resolution digital camera. The chemical characterization of MPs was conducted via FTIR, using a Shimadzu IRTracer-100 spectrometer, covering the infrared range of 400 to 400 cm^–1^. Measurements were conducted with KBr pellets, with each sample undergoing 64 scans at a resolution of 4 cm^–1^. The obtained spectra were compared with reference spectra of known polymers to identify the polymer type. The methodological flowchart is presented in Figure S1. During the MPs analysis, the workspace was carefully cleaned before and after sample handling to minimize contamination risks. The laboratory was kept with windows closed and natural-fiber clothing and wore cotton robes was used for the team during the whole procedure.^39^ Restricted access to personnel during procedures was implemented in the lab routine to reduce the chance of external MP contamination. Additionally, samples were kept covered with aluminum foil when not in use, and experiments were conducted as quickly as possible to avoid environmental exposure. The use of plastics was avoided at every stage, ensuring the reliability and integrity of the results. To minimize cross-contamination in the extraction steps, all glassware and metal was previously cleaned with ultrapure water and covered with aluminum foil and stores and closed boxes.^39^

#### 2.4.2 Analysis of Granulometry

The granulometric analysis of sediments was conducted using classical sedimentology methods.^40^ Initially, sediment samples were placed in an oven at 60 °C for 48 h. Next, 100 g aliquots were taken for wet sieving, using a sieve with a mesh size of 0.062 mm. The fraction passing through the sieve, composed of silt and clay (mud), was collected and subjected to a decantation process lasting 48 to 120 h. The fraction retained on the sieve was dried in an oven at 60 °C for 72 h. This portion was then subjected to dry sieving using a column of sieves stacked together. The particles were agitated for 10 min, and the sediment was separated into fractions retained in each sieve. Each fraction was weighed using an analytical balance. The methodological scheme is shown in Figure S2.

#### 2.4.3 Total Organic Carbon (TOC) Analysis

The quantification of total organic carbon (TOC) was performed using the wet redox volumetric method of organic matter, based on the modified Walkley–Black method.^41^ Initially, 1 g of the sediment sample was transferred to a 500 mL Erlenmeyer flask. Then, 10 mL of 0.5 mol L^–1^ potassium dichromate (K_2_Cr_2_O_7_) solution was added, followed by 20 mL of concentrated sulfuric acid (H_2_SO_4_), promoting the oxidation of organic carbon. The solution was stirred and left to rest for 30 min to ensure complete reaction. After this period, 200 mL of distilled water, 10 mL of 85% orthophosphoric acid (H_3_PO_4_), and 1 mL of 1% diphenylamine indicator were added. The resulting solution was titrated with 0.125 mol L^–1^ ammoniacal ferrous sulfate solution until the color changed from purple to green. The methodological flowchart is detailed in Figure S3.

#### 2.4.4 Extraction and Quantification of Humic Acid (HA)

The procedure for obtaining and quantifying HA was performed following a modified protocol by Chaitra et al.^42^ Initially, 25 g of the sediment sample was transferred to a 50 mL centrifuge tube. Subsequently, 20 mL of NaOH 0.1 mol L^–1^ was added, manually stirred, and left to rest for 24 h. The mixture was then centrifuged at 5000 rpm for 30 min, and the supernatant was collected into a 50 mL beaker. Another 20 mL of 0.1 mol L^–1^ NaOH was added to the centrifuge tube, manually stirred to resuspend the precipitate, rested, and centrifuged again. The resulting supernatant was combined with the previously collected one, yielding an alkaline extract at pH 13. The pH of the extract was adjusted to 1 by adding drops of 20% H_2_SO_4_, followed by an 18 h resting period. The mixture was then vacuum-filtered, and the filtrate was collected and brought to 50 mL with distilled water, resulting in fulvic acid (FA) fractions. The precipitate retained on the filter was washed with 0.1 mol L^–1^ NaOH, and its volume was adjusted to 50 mL with distilled water, yielding the HA fraction. The methodological flowchart is detailed in Figure S4. After obtaining the two fractions, HA content was determined as follows: an aliquot of 5 mL of the HA solution, 1 mL of K_2_Cr_2_O_7_, and 5 mL of concentrated H_2_SO_4_ were transferred to a tube. The mixture was heated to 150 °C for 30 min, then transferred to a 125 mL Erlenmeyer flask and titrated with 0.0125 mol L^–1^ ammoniacal ferrous sulfate solution using ferroin as the indicator under constant stirring. The methodological flowchart is detailed in Figures S4 and S5.

#### 2.4.5 Principal Component Analysis (PCA)

PCA is a statistical method used to reduce the dimensionality of data while preserving the original information. This approach organizes collected data based on observed similarities and differences.^43^ The results are displayed in a two-dimensional space, enabling the identification of similarity patterns among samples. In this type of graphical representation, the proximity of points reflects the similarity in sample properties, facilitating the understanding of their relationships.^44,45^ The analysis included sampling locations (P1 to P9), types (fragments, granules, films, and fibers), and colors of MPs (blue, white, yellow, black, pink, green, orange, and transparent). Additionally, environmental parameters commonly found in estuarine environments were considered, such as organic carbon content, organic matter, humic acid, and granulometry (silt, gravel, and sand). PCA was conducted using the R Project software with the FactoMineR and FactoshinyR packages.

#### 2.4.6 Computational Methods

All molecular dynamics (MD) simulations were performed using GROMACS software^46^ version 2023.2, implemented with the Amber 14sb force field.^47^ The Amber Force Fields have been applied to parametrize similar systems in previous studies.^28,48^ Polyethylene (PE), polypropylene (PP), and polystyrene (PS) were selected as representative MPs, based on their high prevalence in the estuarine environment, as confirmed by FTIR analysis conducted in this study. Additionally, the presence of these polymers is frequently highlighted in environmental studies.^3,49^ However, the interaction mechanisms between HA and these three polymers remain not fully explored. Three boxes with dimensions of 10 nm × 10 nm × 10 nm were constructed for the HA-PE, HA-PP, and HA-PS systems. Each box contained a single polymer type (PE, PP, or PS), with 10 MP molecules and 10 HA molecules inserted. Additionally, each system included approximately 32,000 water molecules and 30 Na^+^ ions, ensuring electroneutrality and neutral pH conditions. Table S3 shows the number and the components present in each system. The HA structure was adapted from the Temple–Northeastern–Birmingham (TNB) model,^50^ which is widely used in MD studies involving humic substances. The model includes three carboxylic groups, five carbonyl groups, two phenolic groups, two amine groups, and four alcohol groups. The molecular structures of HA and MPs used in the study are presented in Figure 2.

**Figure 2:**
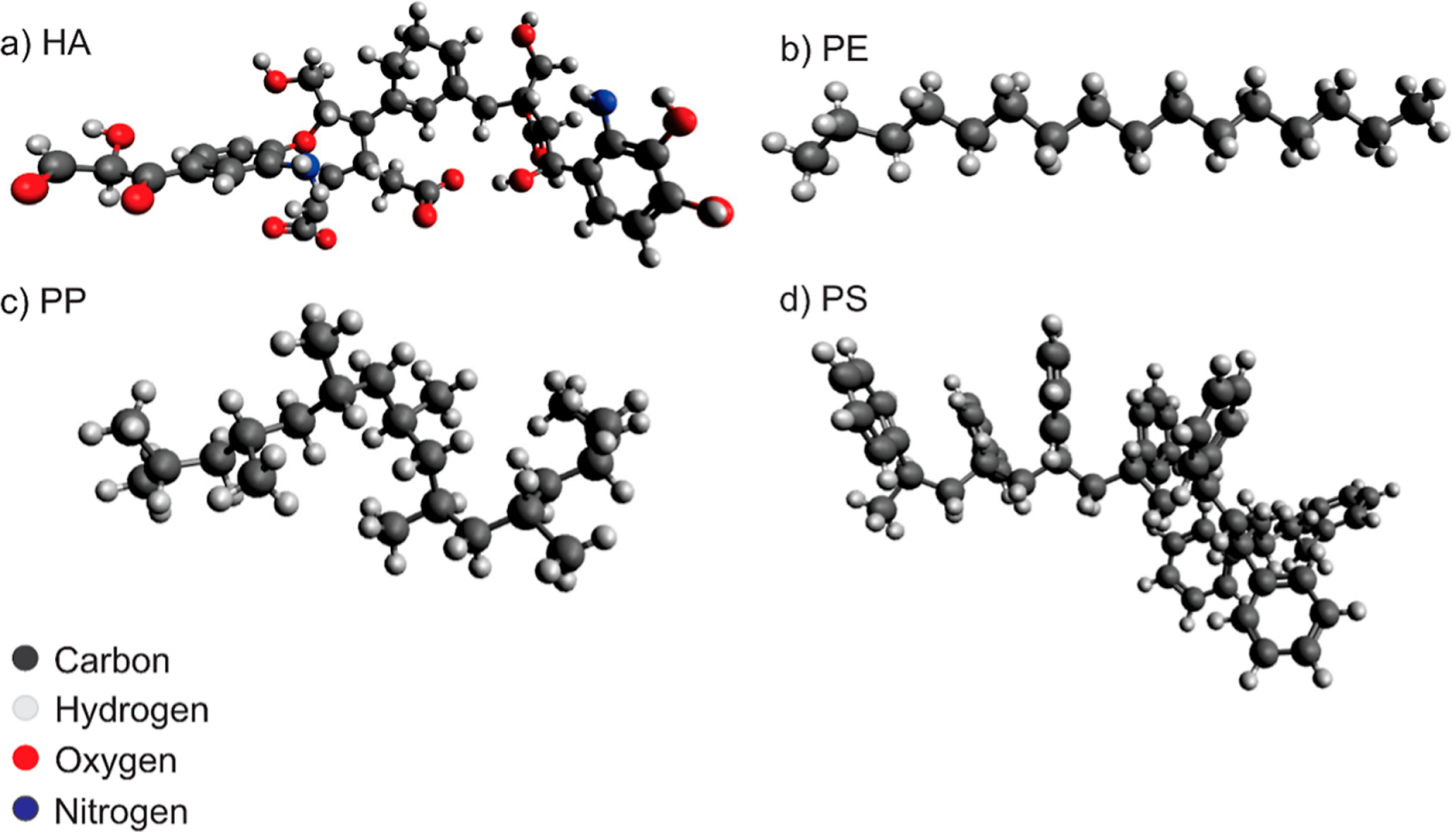
Optimized structures used in MD simulation: HA (a), PE (b), PS (c), and PP (d).

Geometry optimization was initially performed using the Steepest Descent^51^ and Conjugate Gradient^52^ algorithms to stabilize all molecules before the simulation. Subsequently, 200 ps temperature and 100 ps pressure equilibrium steps were performed, using *NVT* and *NPT* ensembles, respectively. The temperature equilibrium was performed using the V-rescale thermostat^53^ at temperatures of 300 K for each system. For the pressure equilibrium, the system pressure was controlled using Parrinello–Rahman barostat^54^ at a value of 1.0 bar. The electrostatic interactions were treated using the particle mesh Ewald (PME) method^55^ with a cutoff of 1.0 nm, which was the same as the van der Waals cutoff. Finally, the production step was performed through a leapfrog algorithm,^56^ for 200 ns, enabling MD and to integrate the equations of motion. The simulation box size (10 nm × 10 nm × 10 nm) and the selected number of molecules (10 MP and 10 HA molecules) were chosen to ensure contact phenomena that could not be well characterized if the concentration of the simulated systems were representative of the real systems, which are dilute. These simulations were performed in a simplified aqueous system to isolate and investigate molecular-level interactions between the polymers and the HA component. This approach did not consider salinity gradients, multivalent ions (e.g., Ca^2+^ and Mg^2+^), pH variability, or sediment mineral surfaces. The inclusion of greater environmental complexity can be explored in future studies.

After the simulations, graphs of the root mean square deviation (rmsd) and interaction potential energy (IPE) were plotted. The rmsd analysis was used to corroborate the convergence of the MD simulations. Furthermore, the independent gradient model (IGM) analysis was performed. The IGM analyses were carried out using the Multiwfn 3.8 software,^57^ and molecular visualizations were generated with the Visual Molecular Dynamics (VMD) program.^58^

## 3 Results and Discussion

### 3.1 Qualitative and Quantitative Analysis of MPs and Chemical Characterization

The results obtained through optical microscopy indicated the presence of MPs with various sizes and shapes at all sampling points, totaling 490 plastic particles. The distribution of MPs per kilogram of dry weight of sediment sampling points is presented in Figure S6. The abundance varied from 16.6 to 363 MPs per kilogram of dry sediment. Notably, the highest number of MPs was observed at sampling points 3 and 4 (Cofeco Wall and Barra Rio Pacoti Beach, respectively). These locations, situated near the ocean and marked by a growing number of residential developments and increasing tourist activity, may explain the significant presence of MPs in these areas. Factors like the local sedimentation parameters and domestic sewage discharge might be attributed by abundance of microplastics.^59^

On the other hand, points 7 (CE-025 Bridge) and 8 (CEAC/Labomar-UFC), situated farther from the ocean and closer to the river, also recorded notable amounts of MPs. This may be attributed to the presence of nearby trails, which can lead to increased plastic waste due to improper disposal by visitors. Additionally, food stalls in the vicinity may contribute to waste generation, which can be carried to these points, adding to the observed plastic pollution. These results underscore the importance of continued monitoring of MPs to better identify sources and distribution patterns in this ecologically significant region.

Furthermore, the colors of the collected MPs were analyzed, as shown in Figure S7. A variety of colors was observed, including white, transparent, blue, pink, black, yellow, green, and orange. The diversity in color reflects the multitude of sources contributing to their introduction into the environment.^60,61^ A closer examination of the points revealed that point 8 (CEAC/Labomar-UFC) exhibited the highest total number of MPs, with a notable predominance of yellow particles, a value significantly higher compared to the others. This result highlights the need for targeted studies to identify the sources linked to the occurrence of this color. The intense presence of people near these points may be compromising the environment. In addition to color, the morphological characteristics of the MPs were analyzed, as illustrated in Figure S8. The results regarding the types of MPs showed that films accounted for 83.05% of the total, followed by fragments (9.21%), fibers (4.39%), and granules (3.35%). The prevalence of films is associated with the use of thin single-use plastic bags, which are widely used in daily life.^62^ The lightweight nature of films facilitates their transport over long distances, enabling widespread dispersion.^63^ Fragments result from the degradation of bottles and plastic packaging into smaller particles over time, while fibers originate from the shedding of clothes and textiles during use or washing, as well as from fishing nets.^68^ Lastly, the presence of granules is linked to personal care or cosmetic products.^63,64^ Furthermore, chemical characterization was performed using FTIR, revealing the predominant presence of three types of MPs (PE, PS, and PP). PE is commonly found in plastic bags,^65^ PS is typically present in disposable cups and food packaging,^66^ and PP is associated with clothing and food packaging.^67^ The presence of these types of MPs is frequently reported in studies on estuaries. For instance, the predominance of PE and PP was reported in the Rio Formoso estuary, Pernambuco, Brazil.^68^ Similarly, PP particles were identified in sediment samples from the Rio de La Plata estuary, Argentina.^69^ In the Rio Cocó estuary, Ceará, Brazil, the occurrence of PE, PP, and PS MPs was observed.^70^ The recurrence of these types of MPs in various matrices, including estuarine sediments, highlights the need for further studies on their sources, dynamics, and ecological impacts.

### 3.2 Granulometric Analysis and % OM, % OC, and % HA

Given the complexity of environments, the sediments were characterized using well-established experimental methods to understand their distribution across different locations. The characterization included commonly studied parameters in sediment research,^71^ such as granulometric fractions (gravel, sand, and mud), organic matter (OM), organic carbon (OC), and humic acid (HA) content. These sediment parameters can influence the tendency of interaction between HA and MPs, necessitating their consideration. The results of the analysis are presented in Table S3. The findings revealed high sand content across the studied sites, which is associated with the proximity to the beach zone, favoring sand deposition. In almost all locations, except for Point 1 (Cofeco Estuary), the muddy fraction was predominant compared to gravel. This sediment profile, characterized by the predominance of sand and mud, aligns with studies in estuarine environments.^71,72^ Regarding organic components (%OC, %OM, and %HA), the highest levels of HA were recorded at points 3 (Cofeco Wall) and 4 (Barra Rio Pacoti Beach). These values may be influenced by factors such as domestic sewage, leaching from urbanized areas, and petroleum combustion.^73^ The higher concentration of HA in these locations aligns with significant amounts of MPs found in the same areas, suggesting that this organic fraction could be influencing the accumulation of MPs in estuarine sediments. Based on these results, statistical tools such as PCA were applied to further investigate this association. PCA is widely used to explore correlations among environmental variables,^74^ making it particularly suitable for complex systems and providing deeper insights into the behavior of MPs in estuarine environments.

### 3.3 Principal Component Analysis (PCA)

Humic fractions, especially humic acids, are present in the composition of the sedimentary matrix.^75^ Due to the molecular complexity (functional groups), it is clear the existence of the interaction with microplastics. Additionally, this environmental correlation can influence the sorption parameters of microplastics and contaminants, affecting their environmental mobility.^76^ Therefore, interaction studies between humic acids and microplastics is important to understand the environmental dynamic of microplastics in natural sediment. In this study, the principal component analyses (PCA) were used to identify and classify the correlations of humic acids (HA) and microplastics (MP) in the different depositional areas of the Pacoti River (surface fluvial sediments) (Figure 2).^77^

The results showed that the first principal component (PC1) accounted for 47.30% of the variance, and the second principal component (PC2) explained 23.01%, representing 70.31% of the total data set variance, as shown in Figure 3, suggesting that most of the information on all initial variables analyzed was validated.

**Figure 3:**
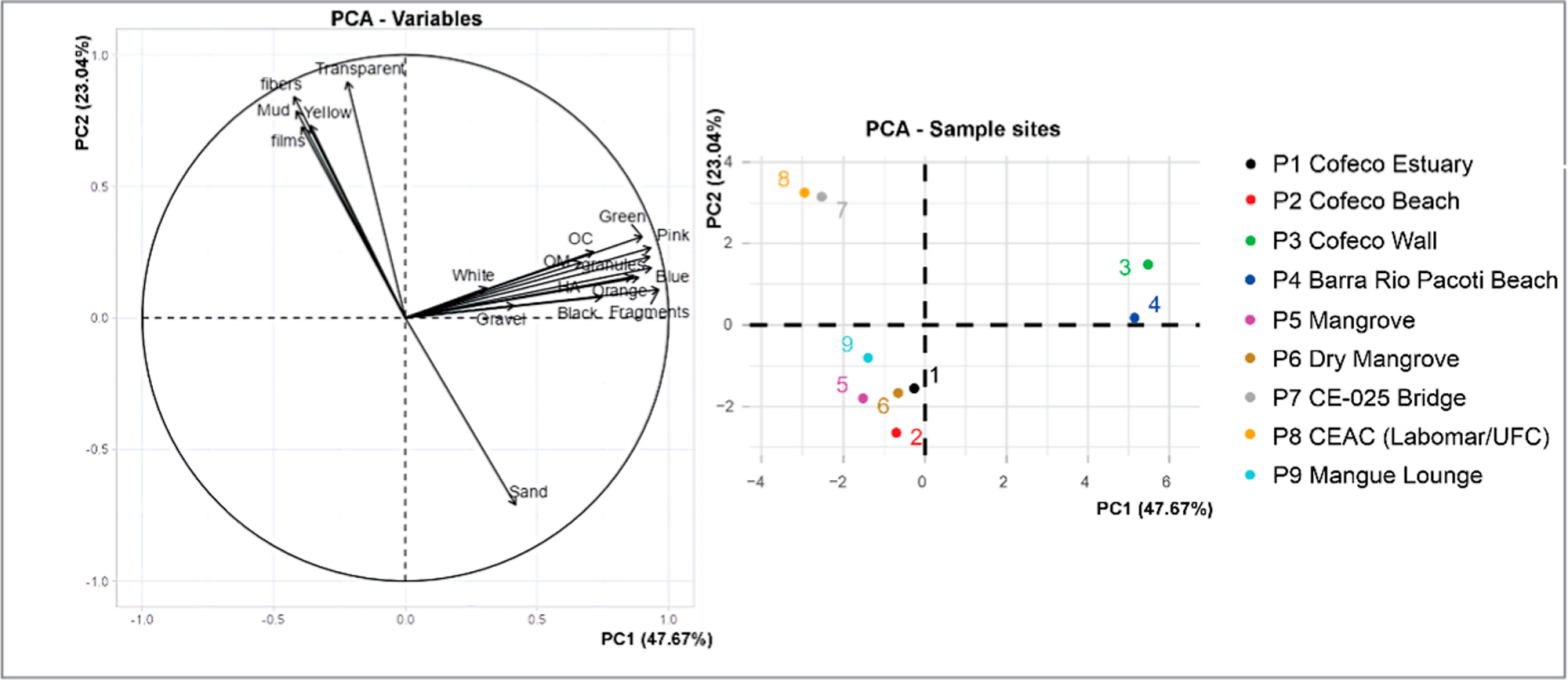
PCA analysis of the variables and sampling sites.

The results obtained from PC1 (47.30%) evidenced a positive correlation between sampling points 3 (Cofeco Wall) and 4 (Barra Rio Pacoti Beach), which exhibited higher levels of humic acid (HA) and different types of microplastics (fragments, granules), along with a wide variety of colors (green, black, blue, orange, pink, and white). Similar results were observed in correlation studies where humic fractions showed preferential adsorption on microplastics.^77^ Furthermore, factors such as high molecular weight and the degree of humification affect the degradation of microplastics in riverine environments, due to the presence of aromatic rings and quinone groups in their molecular structure.^78^ The PC2 (23.01%) revealed a positive correlation between sampling points 7 (CE-025 bridge) and 8 (CEAC/Labomar-UFC), associated with fibers and films of yellow and transparent coloration, highlighting the deposition of MPs in these areas.

The distance to the coastline may be one of the factors that influence the microplastics to the humic acid deposited in the sediment. The samples near coast line (P3 and P4) was grouped with different microplastics detected in this study, and located in the right area of the coordinate, which was positively contributed by PC1. In contrast, the samples far from the coast line (P7 and P8) were grouped and located in the left area of the coordinate, which was negatively contributed by PC1. This indicates that the distance from the coastal zone can be affecting depositional processes of the river sediments and microplastics are dominant. Another factor affecting this HA-MP interaction is the depth of the area. Studies indicated that at greater depths, stronger HA-MP correlations are observed compared to the surface areas analyzed.^77^

Laboratory-scale studies also show a clear trend in HA adsorption onto PM, altering its shape and chemical composition, directly influencing ingestion by organisms^79^ and the potential for transporting other contaminants.^30^ However, the correlation between HA and MPs is not fully clear in fluvial sediments and formal adsorption studies are required to understand this interactions.^77^

Considering that the chemical structures of both MPs and HA are well-known, the application of computational approaches, such as molecular dynamics simulations, emerges as an interesting strategy to deepen the understanding of these interactions at the molecular level. Although such approaches are widely used in studies evolving MPs,^29,33,34^ their application to investigate HA-MP interactions remains underexplored. The urgency to comprehend these interactions becomes even more relevant in estuarine environments due to their ecological importance and the constant presence of HA in these regions.^80,81^

It is important to emphasize that the correlations identified by PCA do not necessarily imply direct causality between humic acids and microplastics. Estuarine systems are influenced by multiple environmental variables acting simultaneously, including hydrodynamic conditions, granulometry, depositional energy, tidal influence, proximity to the coastline, organic matter availability, and anthropogenic inputs. Therefore, the observed HA-MP associations should be interpreted as indicative of possible environmental relationships within a complex multivariate system rather than as evidence of direct cause-and-effect interactions.

### 3.4 Molecular Dynamics Simulations

The environmental observations and PCA analysis suggested possible associations between humic acids (HA) and different classes of microplastics (MPs) in the estuarine sediments, particularly involving polyethylene (PE), polypropylene (PP), and polystyrene (PS) identified in the studied areas. Although PCA provided important evidence of environmental correlation patterns, the molecular-level mechanisms governing these associations could not be directly inferred from the field data alone.

Therefore, molecular dynamics (MD) simulations were employed as a complementary approach to investigate the intermolecular interactions between HA and the selected MPs under controlled conditions. The computational models were designed to evaluate whether the interaction trends suggested by the environmental data set could be supported by energetic, structural, and noncovalent interaction analyses, thereby providing mechanistic insights into the HA-MP association processes observed in the estuarine environment.

The molecular structures of the systems were constructed and initially subjected to energy minimization, allowing for the adjustment of atomic positions and reduction of the potential energy, thereby ensuring stable starting configurations for the simulations. Following the production runs, the structural stability of each system was evaluated through the root mean deviation (rmsd) plotted against simulation time (Figure 4). This analysis allowed for the assessment of structural fluctuations and the stability of the systems throughout the simulation period. In addition, the convergence criterion in this result is based on the observation that the rmsd values did not exhibit large deviations during the production run.

**Figure 4:**
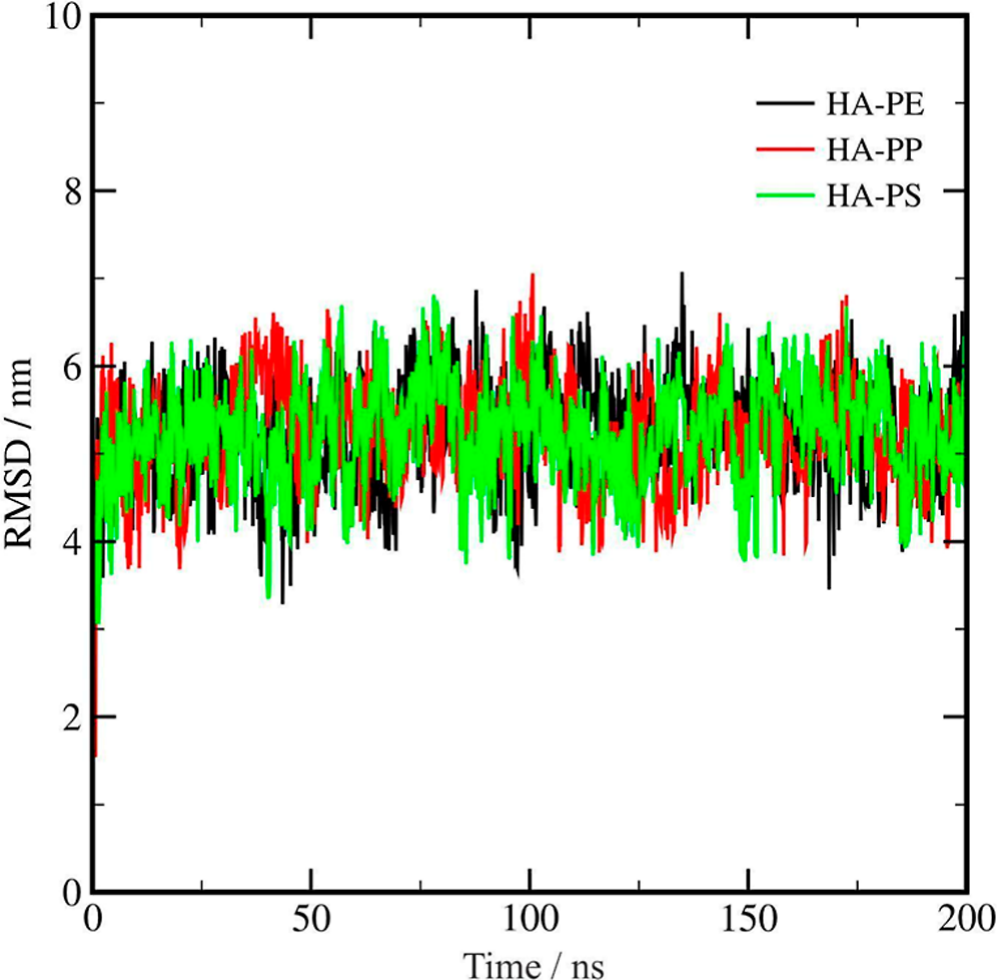
Root mean deviation (rmsd) plots versus time.

The rmsd graph revealed minimal and regular fluctuations around the average values for each system (HA-PS = 5.22 ± 0.05 nm; HA-PE = 5.25 ± 0.04 nm; HA-PP = 5.24 ± 0.06 nm). The maintenance of these values throughout the simulation indicates that the structural configurations of the MPs and HA molecules remained stable over time, exhibiting only minor structural deviations. These results demonstrate that the interaction between MPs and HA was stable and persistent during the simulation period. Therefore, the 200 ns time for the production step was chosen once it was sufficient to allow the systems to reach thermodynamic equilibrium. As shown in the rmsd graphs (Figure 4), in which large deviations were not observed during the production step, the structural configurations of MP and HA molecules remained stable over time. It confirms that the time chosen for the simulation effectively captured the interactions in a stationary state.

To further investigate these interactions, the IPE was evaluated throughout the entire simulation (0–200 ps). The IPE is used to quantify the interaction strength between HA and MPs (PE, PP, and PS) by summing the contributions of van der Waals and electrostatic energies. Figure 5 illustrates the IPE values, highlighting the interaction strength between HA and each MP type.

**Figure 5:**
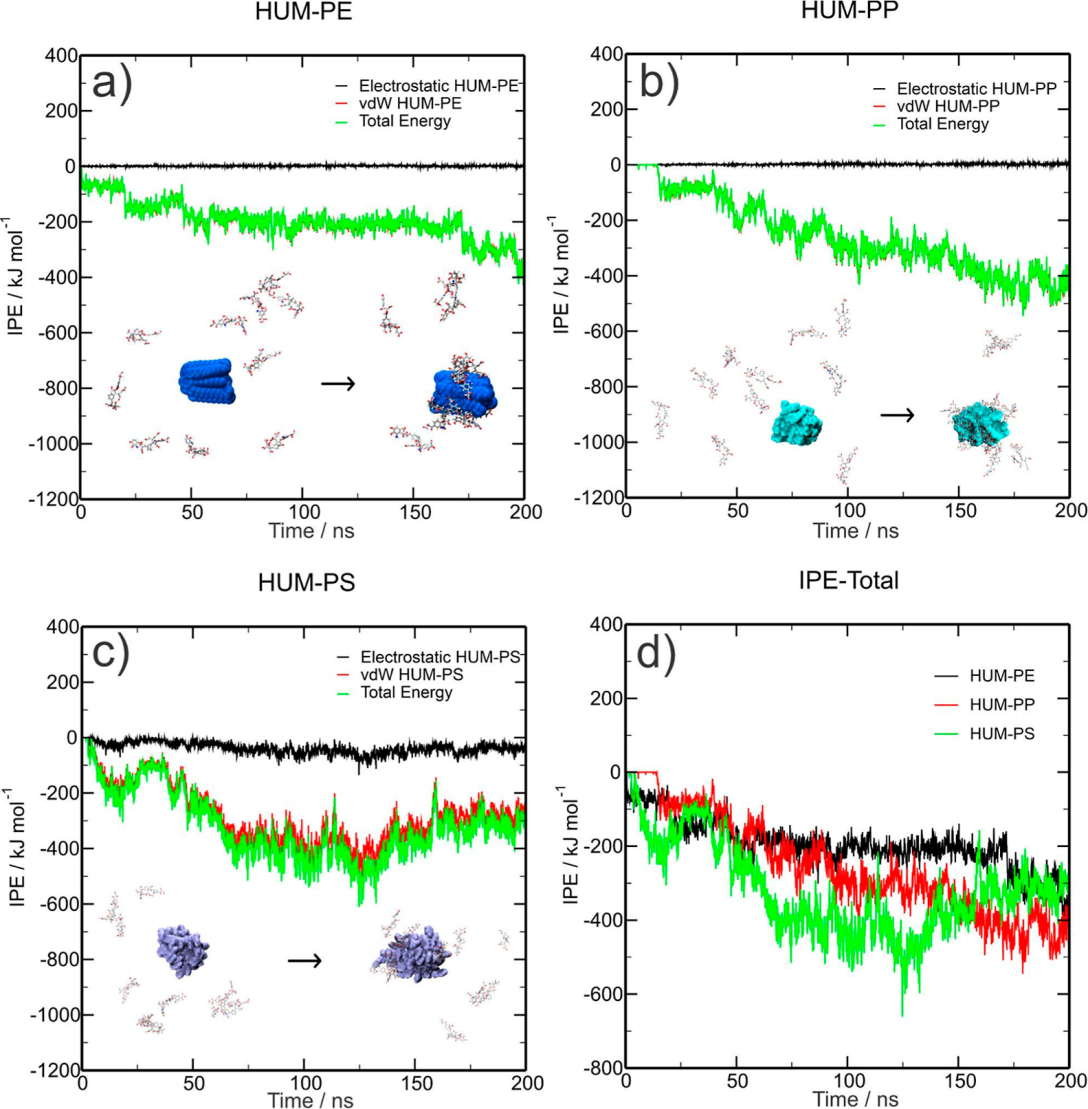
IPE for HA-MP systems over the 0–200 ns simulation: HA-PE (a), HA-PP (b), and HA-PS systems (c). Comparison of total IPE among the three MP types (d).

In all systems, it was initially observed that the MPs and HA were spatially separated. The HA molecules were dispersed throughout the simulation box and, over the course of the simulation, established interactions with the surfaces of the MPS. The results suggest a spontaneous approach between HA and the MPs, resulting in the formation of stable structures. The systems were predominantly stabilized by van der Waals interactions, with a smaller contribution from electrostatic forces. These findings are in line with previous studies, which also emphasized the key role of van der Waals interactions in the stabilization of systems involving PE, PP, and PS with different molecular species.^12,29,33,34^

The interaction energy profiles reveal that, in the HA-PE system (Figure 5a), van der Waals interactions (red line) were predominant, while electrostatic contributions (black line) approached zero throughout the simulation. A similar behavior was observed for the HA-PP system (Figure 5b), also characterized by dominant van der Waals interactions and minimal electrostatics. The HA-PS system (Figure 5c) demonstrated a more significant electrostatic contribution compared to the other systems, although van der Waals interactions still represented the main interaction mechanism. Following the analysis of individual interaction components, the total IPE between HA and each MP was evaluated over the simulation time (Figure 5d). The HA-PS system exhibited the lowest total interaction energy values (Figure 5d), indicating stronger overall interactions compared to HA-PP and HA-PE. A quantitative assessment of the average total energy revealed values of −316 ± 51 kJ mol^–1^ for HA-PS, −195 ± 27 kJ mol^–1^ for HA-PE, and −254 ± 63 kJ mol^–1^ for HA-PP, suggesting a higher affinity between HA and PS. The IPE analysis reflects the thermodynamic stabilization of the formed complex. More negative values indicate a higher energy release to the environment, which is physically translated to stronger attractive forces overcoming the repulsive forces. While all the systems are predominantly governed by van der Waals’ dispersions, the PS differs from PE and PP for having aromatic rings in its monomers. Once the HA structure is also rich in aromatic domains, the HA-PS approximation allows the stacking and overlap of electron density, promoting strong noncovalent interactions. El Gaayda et al.,^82^ analyzed the electron distribution in HA and found that the highest occupied molecular orbitals (HOMO) are mainly concentrated in the aromatic rings of aromatic carboxylic acids. This finding was also suggested by Zhang et al.,^48^ who proposed that pi–pi interactions may play an important role during the interaction process involving HA. Therefore, the interaction energies’ magnitude obtained in the simulations corroborate quantitatively what was proposed by El Gaayda et al.^82^ showing numerically that the noncovalent interactions, particularly the pi-stacking, are the main responsible parties for the higher affinity observed between the PS and HA.

After the energetic analysis, the number of hydrogen bonds between HA and water molecules was evaluated for each system (Figure 5). Humic acid contains oxygen functional groups with hydrogen bonding capability.^26^ The hydrogen bond was considered to be present when the distance between an oxygen atom of a carboxyl group in HA and the oxygen of a water molecule was less than 3.5 Å, and the angle was less than 30°.^12^ The graph of the number of hydrogen bonds over time is shown in Figure 6.

**Figure 6:**
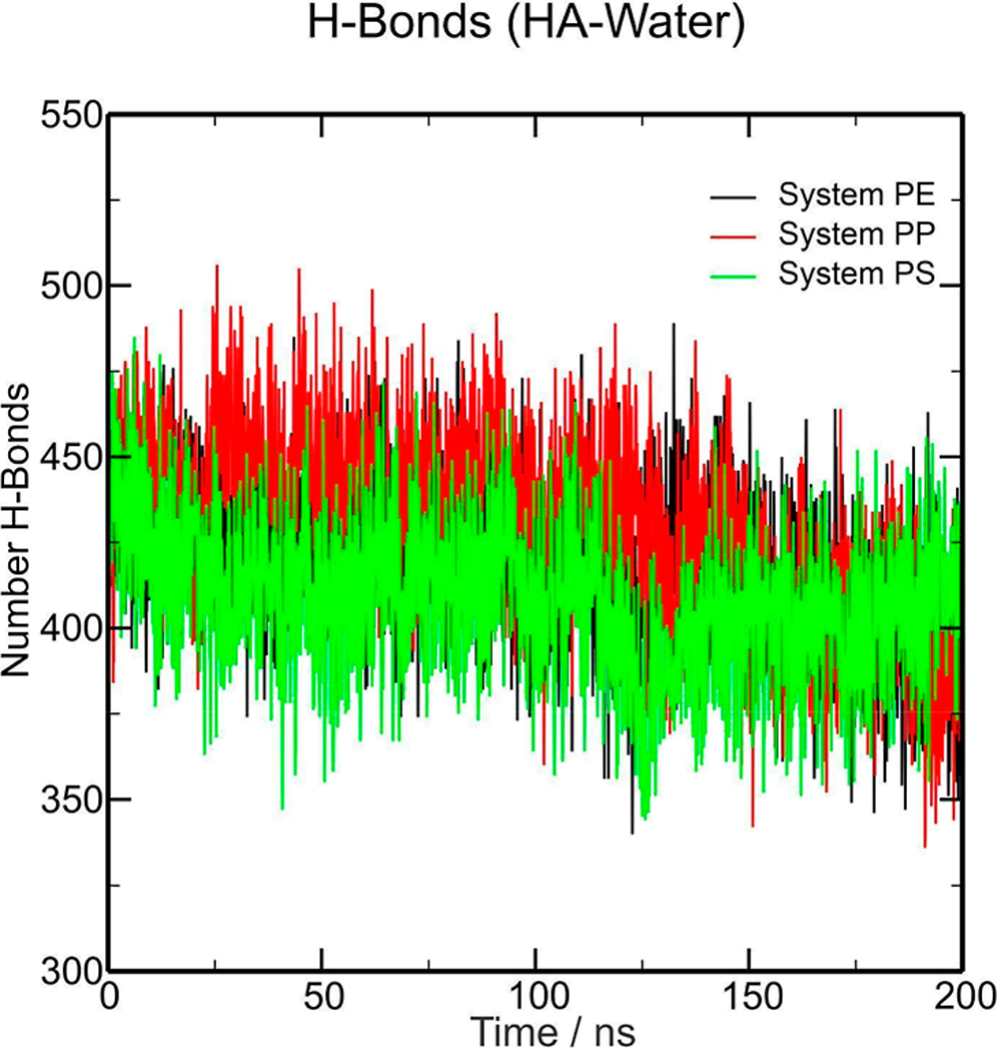
Time evolution of the number of H-bonds (HA-water).

The results show that, as the simulation progresses (Figure 6), the number of hydrogen bonds between HA and water gradually decreases. Initially, HA forms numerous bonds with water molecules. However, over the course of the simulation, a reduction in these interactions is observed due to the contact with MPs present in the system. This behavior change suggests the establishment of interactions between HA and MPs, as supported by the IPE. Notably, in the HA-PS system, a more significant decrease in the number of hydrogen bonds between HA and water was observed in comparison to the PE and PP systems (Figure 6). This behavior suggests a reduced exposure of HA to water, likely due to its interactions with PS. Such behavior reflects an affinity between HA and PS, consistent with trends observed in the EPI analysis. Based on the findings obtained, which suggest that the interaction between HA and the MPs is primarily influenced by weak intermolecular interactions, such as van der Waals forces. The IGM (independent gradient model) was applied to confirm the suggestion. The IGM calculations were performed to analyze the distribution and nature of noncovalent interactions.^83^ The eqs 1 and 2 are used to obtain the IGM function (δ*g*). The function IGM (δ*g*) = *g* ^IGM^(*r*) – *g*(*r*)^34,72^ where

1 $$g\left(r\right)=\left|\sum_{i}{{\Delta \rho }_{i}}^{free}\left(r\right)\right|$$

2 $$g^{IGM}\left(r\right)=\sum_{i}\left|\Delta {\rho_{i}}^{free}\left(r\right)\right|$$

The function δ*g* is obtained from gradient of electron density, with *i* being the index of atoms. Similar to the NCI method, the IGM maps use the sign λ_2_ρ­(*r*) to distinguish the characteristics of revealed interactions. The λ_2_ρ­(*r*) < 0 indicate the strongly attractive, like hydrogen bonds, when λ_2_ρ­(*r*) ≈ 0 represents the van der Waals interactions, and values of λ_2_ρ­(*r*) > 0 repulsive interactions such as steric hindrance.^83^ The IGM maps are shown in Figure 7.

**Figure 7:**
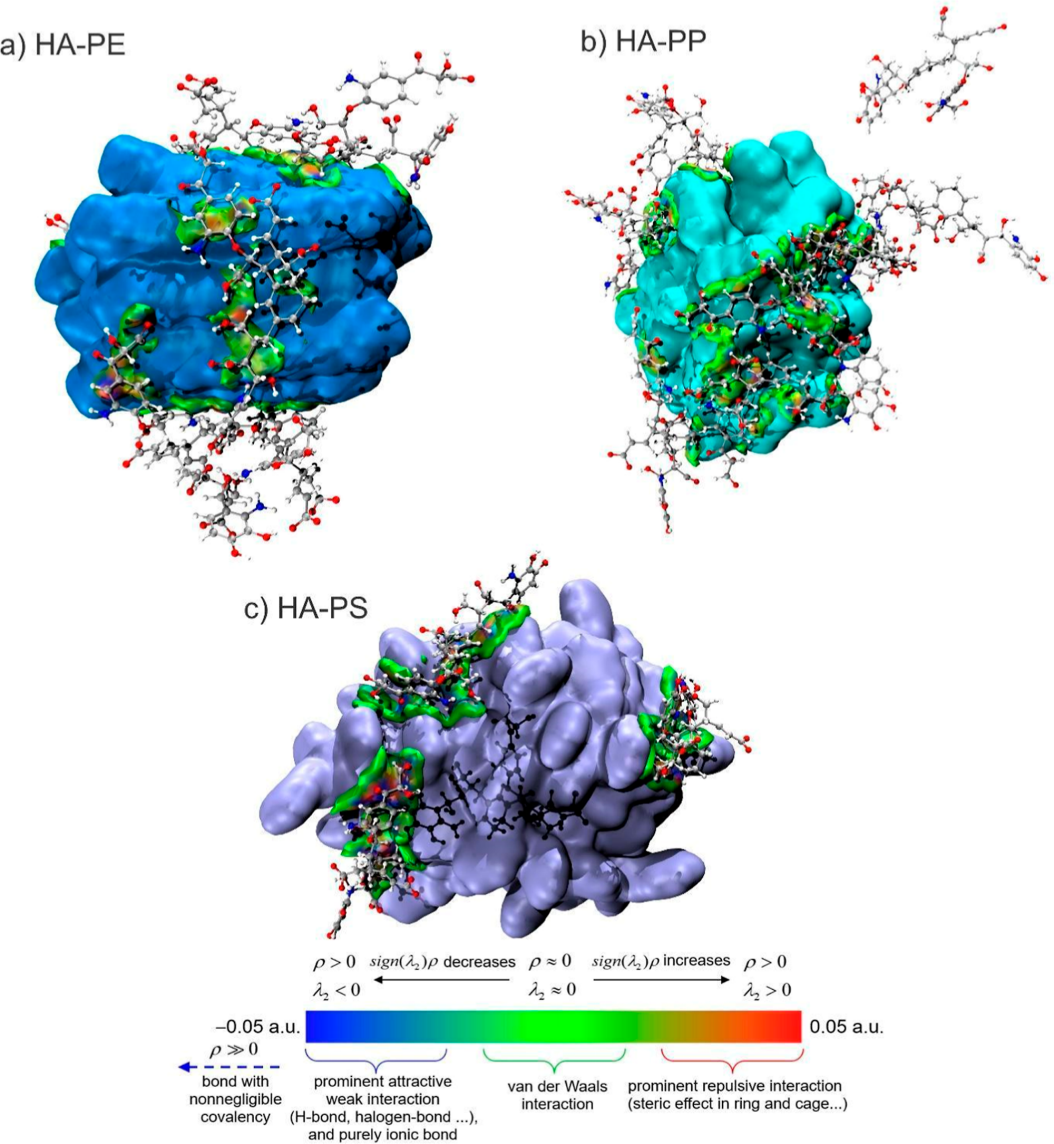
IGM isosurface map of HA-PE (a), HA-PP (b), and HA-PS (c).

For the analyses, the coordinates of the last frame of the molecular dynamics were selected, calculating the promolecular electron density, considering all the influences of solvent interaction forces over the trajectory time. Knowing this, only PE, PP, PS, and HA fragments were selected for better visualization and analysis, in order to evaluate only the nature of the intermolecular interactions between the MP-HA pairs. Additionally, the HA-PS (Figure 7c) system exhibits a more intense presence of blue regions compared to the other systems, HA-PE (Figure 7a) and HA-PP (Figure 7b), indicating stronger attractive interactions. This is information particularly evident near the oxygen atom in the HA structure (represented by the red atom), as shown in Figure 7c.

As shown previously in Figure 6, HA-MP interactions increase, mainly for PS, indicating a preferential interaction with this MP. PE has chains of CH_2_– sequences that dominate hydrophobic interactions and can influence the organization between PE chains, thus decreasing interactions with HA. In PP, there are –CH_3_ groups in its monomeric structure, which, when agglomerated, create cavities that can favor interactions with HA through their hydroxyl, carbonyl, and amine groups. PS has aromatic rings in its monomers; therefore, its interaction is dominated by aromaticity. Its agglomeration can favor interactions with HA through pi-stacked stacking of the aromatic groups of PS and HA. This result is also aligned with the IPE values, reflecting a greater affinity between these molecules compared to HA-PE and HA-PP. These observations are further corroborated by the IGM analysis (Figure 7c), which shows a preference for interaction with PS. These interactions are reinforced by stronger van der Waals forces, which can resemble hydrogen bonds in terms of distance and strength. This behavior can be observed by the greater proximity between HA molecules in regions of the PS cavities, which favors stronger local interactions. This finding is a significant contribution of this work, as previous studies have not explicitly demonstrated the preferential interaction with PS, nor provided such a clear visualization of both van der Waals interactions.

### 3.5 Environmental Implications

The interaction of microplastics with humic acids has implications for the transport processes. A strong affinity between HA and MP, as observed in the HA-PS system, suggests the possibility of the developing an eco-corona on the plastic surface in the environmental context.^84^ The aggregation of organic matter increases the size and density of MPs, thereby increasing their settling velocity and facilitating the deposition in the sediment bed.^85^

The consequence of this is that, after the MPs are deposited in the sediment due to the density, they come to represent a long-term source of exposition for benthic organisms. These organisms, like mollusks, are highly prone to bioaccumulate contaminants. The massive deposition of these MPs, enriched with organic matter, such as HAs, directly into their habitat is dangerous to human health, as some of these organisms serve as commercial food.^86^

Additionally, humic acids are heterogeneous chemical structures composed of aromatic, aliphatic chains, oxygenated functional groups, and variable molecular weight fractions. Although detailed spectroscopic and molecular weight characterization of the extracted HA fractions was beyond the scope of the present study, the interaction mechanisms discussed here are supported by previously established structural models and experimental observations reported in the literature. Future studies employing complementary techniques such as FTIR mapping, fluorescence spectroscopy, XPS, and molecular weight distribution analyses may provide additional insights into the environmental behavior of HA-MP systems.

## 4 Conclusion

In this study, the interaction between humic acids (HA) and microplastics (MPs) was investigated through experimental methods, combined with computational analyses. The experimental results, supported by multivariate statistical analysis (PCA), revealed interaction trends between HA and MPs across different environmental parameters, particularly sediment granulometry (gravel, sand, and mud), humic acids (HA), organic carbon (OC), and organic matter (OM). In this context, computational approaches were employed to further explore these interactions. The results showed that all simulated systems (HA-PE, HA-PS, and HA-PP) were predominantly governed by van der Waals forces, with PS showing the highest affinity for HA. The independent gradient model (IGM) analysis enabled the visualization of van der Waals interactions as well as hydrogen bonding, with a predominance of hydrogen bonding observed in the PS system. This affinity among the three types of MPs represents a potential risk in environmental matrices, particularly in the presence of PS. Furthermore, complementary future studies were necessary, incorporating multitemporal sampling strategies, including different seasons, depth and different environmental parameters, in order to better understand the complexity dynamics of MP interactions and their interactions with humic substances in tropical estuarine environments. However, this study aimed provides molecular-level initial insights into HA-PS interactions in tropical coastal environments of Brazil, recognizing the importance and necessity more efforts focused on understanding and mitigation arising from this interaction in these environments. It is also important to highlight the need for future approaches to conduct a more comprehensive study of the effects of variables such as pH, salinity, and ionic strength, to assess the behavior and influence on the dynamics and interactions of HA-PS in tropical estuaries.

Finally, we recognize the need for further work to corroborate the results described here and to confirm the postulated mechanism of interaction between different humic fractions (humic and fulvic acids) and microplastics. Although the MD model employed was important for isolating and elucidating interactions between HA and MPs, it is recognized that the simplified aqueous system presents limitations compared to the extreme complexity of estuarine environments. The absence of factors like ionic strength, pH variability, and the presence of multivalent ions consists of a methodological choice aiming to avoid interference effects in the interactions of interest. However, aiming to investigate the dynamics of agglomeration and natural deposition theoretically, it is necessary to develop new computational studies that incorporate these variables. In addition, surface analysis techniques (e.g., XPS, FTIR mapping) further research should be conducted to investigate the qualitative and quantitative in different organic matter fractions adsorption, extend to biodegradable and nonbiodegradable microplastics, in order to fill the current gaps in the existing literature.

## Supplementary Material



## Notes

Sample sites information, samples mass, number of molecules and ions present in each system used in MD simulations, characterization of the sediment samples, MPs extraction methodology from sediments, granulometric analysis, total organic carbon analysis, extraction and quantitative analyses, interaction energies for humic acids and microplastics (van der Waals, electrostatic, and total) and additional graphs to provide more details of the microplastics characterization results and improve discussions (PDF)
