# Supplementary material for: Insights About Interactions between Microplastics and Humic Acids: An Approach Theoretical Modeling and Experimental
Source: ACS Omega. 2026 Jul 4;11(28):41304–16. doi: 10.1021/acsomega.5c12106 (PMC13393201; doi:10.1021/acsomega.5c12106)
Supplement: Supplementary file 1 [file ao5c12106_si_001.pdf]

SUPPORTING INFORMATION

Insights about interactions between microplastics and humic acids: An approach theoretical modeling and experimental

Guilherme de S. Parente [†], Leonardo P. da Silva[‖], Lucas L. Bezerra[‖], José Osmar de S. Júnior[‖], Caroline B. Reinaldo[†], Christian P. Almeida [†], Norberto de Kassio V. Monteiro[‖]*, Laercio L. Martins*[a], Andre H. B. Oliveira*†.

[†] Environmental studies laboratory (LEA), Federal University of Ceará (UFC), Fortaleza, Ceará, 60440-900, Brazil.

[‖]Theoretical chemistry group (GQT), Federal University of Ceará (UFC), Fortaleza, Ceará, 60440-900, Brazil.

a Laboratory of Petroleum Engineering and Exploration (LENEP), North Fluminense State University (UENF), Macaé, Rio de Janeiro 27932-125, Brazil

*Corresponding author: E-mail address: norbertokv@ufc.br (N.K.V. Monteiro); laercio@lenep.uenf.br (L.L. Martins); andrehbo@ufc.br (A.H.B. Oliveira)

The Supporting Information includes

Numbers of pages: 9

Numbers of Tables: 5

Numbers of Figures: 8

CONTEXTUALIZATION OF MICROPLASTIC POLLUTION

Microplastic pollution is a global threat to terrestrial and aquatic ecosystems These plastic fragments, ranging in size from 1 µm to 5 mm, result from the fragmentation of larger plastics or originate directly from primary sources, such as textile fibers.[1] They are often associated with human activities, including tourism, fishing, and improper waste disposal, impacting both aquatic and terrestrial environments.[1–3]

Due to their persistence, potential for bioaccumulation, toxicity, and ability to disperse over long distances, microplastics (MPs) have become a major focus of environmental research.[1–4] They have been detected in various environmental matrices, such as sediments and surface waters,[5–8] and organisms,[9–11] revealing their widespread presence and deposition potential. Specifically, in estuarine sediments, studies have shown specific deposition patterns have been reported, often involving interactions with organic matter, especially Humic Acid (HA), which influence their environmental dynamics.[12–14]

The lack of effective strategies to mitigate plastic pollution contributes to the increasing presence of these materials in estuarine environments. Despite local and global efforts aimed at reducing plastic waste, significant challenges remain.

Table S1. Sample sites, location and coordinates of collection points

**Sample sites**	**location**	**Coordinates**
P1	Cofeco Estuary	3°49’40’’S 38 °25’04’’W
P2	Cofeco Beach	3°49’18’’S 38 °24′08’’W
P3	Cofeco Wall	3°48’53’’S 38°24’37’’W
P4	Barra Rio Pacoti Beach	3°49’52’’S 38°23’54’’W
P5	Mangrove	3°49’29’’S 38°24’06’’W

P6	Dry Mangue	3º49’30’’S 38º24’19’’W
P7	CE-025 Bridge	3º49’25’’S 38º25’14’’W
P8	CEAC (Labomar/UFC)	3º50’00’’S 38º25’24’’W
P9	Mangue Lounge	3º50’34’’S 38º25’24’’W

Table S2. Location and dry sediment mass

Location	Sediment mass
Cofeco estuary	420 g
Cofeco Beach	397 g
Cofeco Wall	524 g
Barra Rio Pacoti Beach	341 g
Mangrove	516 g
Dry Mangue	576 g
CE-025 Bridge	518 g
CEAC (Labomar/UFC)	581 g
Mangue lounge	583 g

Table S3. Number of molecules and ions present in each system used in MD simulations

**System PE-HA**	**System PP-HA**	**System PS-HA**
Polyethylene (10)	Polypropylene (10)	Polystyrene (10)
Humic acid (10)	Humic acid (10)	Humic acid (10)
Water (32267)	Water (32214)	Water (31976)
Sodium (30)	Sodium (30)	Sodium (30)

Table S4. Percentage data from granulometric analysis, organic matter content, organic carbon, and humic acid levels found in sediment samples.

Sample sites	Location	%CO	%AH	%MO	%Gravel	%Sand	%Mud
P1	Cofeco Estuary	7.79	0.0022	13.44	7.33	92.36	0.31
P2	Cofeco Beach	5.29	0.0045	9.13	0.04	99.08	0.88
P3	Cofeco Wall	5.99	0.0413	10.33	0.05	88.79	11.16
P4	Barra Rio Pacoti Beach	6.47	0.0321	11.16	0.08	99.53	0.39
P5	Mangrove	5.23	0.0038	9.03	0.01	97.96	2.03
P6	Dry Mangue	5.88	0.0268	10.14	0.03	97.30	2.67
P7	CE-025 Bridge	6.16	0.0091	10.62	0.73	60.31	38.96
P8	CEAC (labomar/UFC)	5.63	0.0084	9.72	0.55	79.19	20.26
P9	Mangue Lounge	5.65	0.0187	9.74	4.16	74.22	21.62

Table S5: van der Waals, electrostatic, and total interaction energies for Humic acid and microplastics (PS, PE, and PP)

Type of interaction	HA-PS (kJ/mol)	HA-PE (kJ/mol)	HA-PP (kJ/mol)
van der Waals	- 278.3 ± 45	- 196 ± 27	- 256 ± 63
Electrostatic	- 37.9 ± 7	1.22 ± 0,24	1.62 ± 0,44
Total	- 316 ± 51	- 195 ± 27	- 254 ± 63

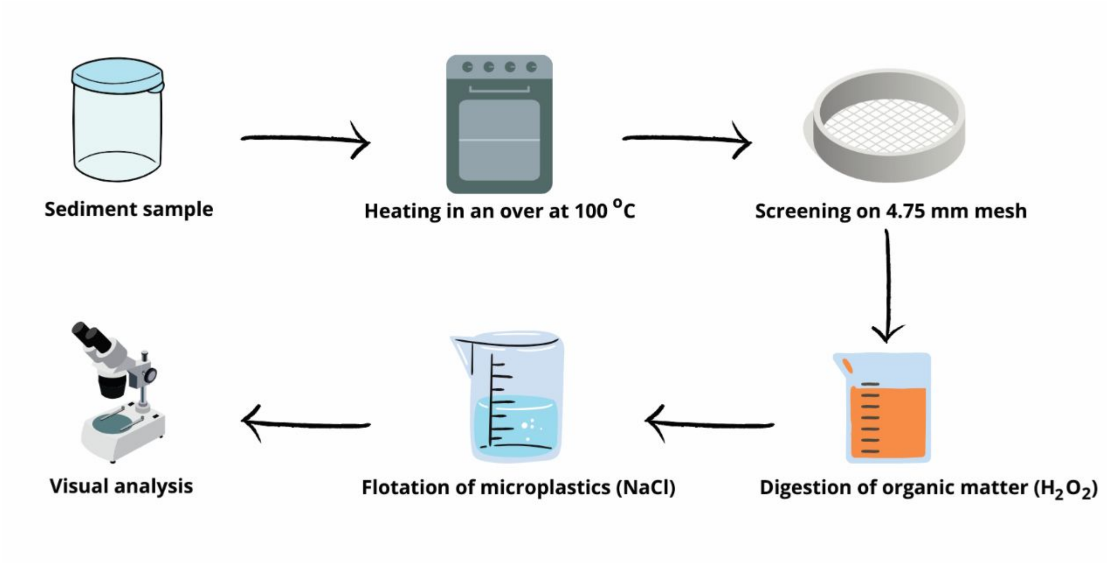

Figure S1. Methodology used to extract MPs from sediments. The procedure was adapted from Akkajit *et al.*[15] and Masura *et al.*[16]

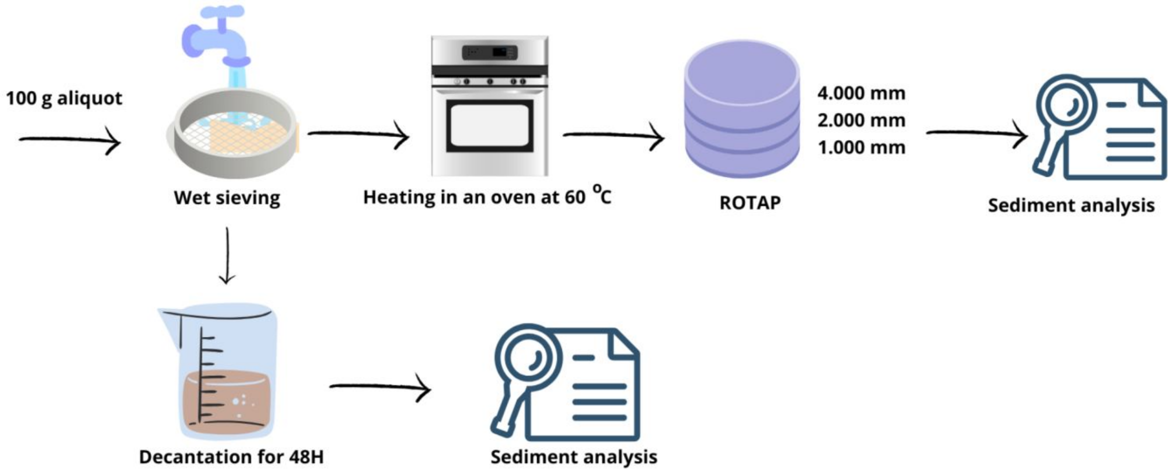

Figure S2. Methodology used for granulometric analysis was adapted from Harris.[17]

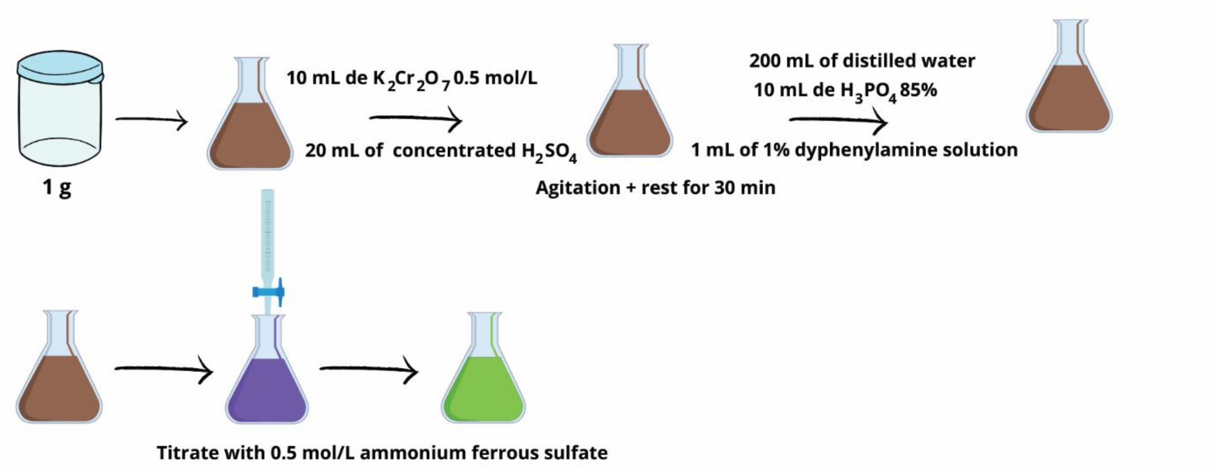

Figure S3. Methodology used for total organic carbon analysis based on the modified Walkley Black.[18]

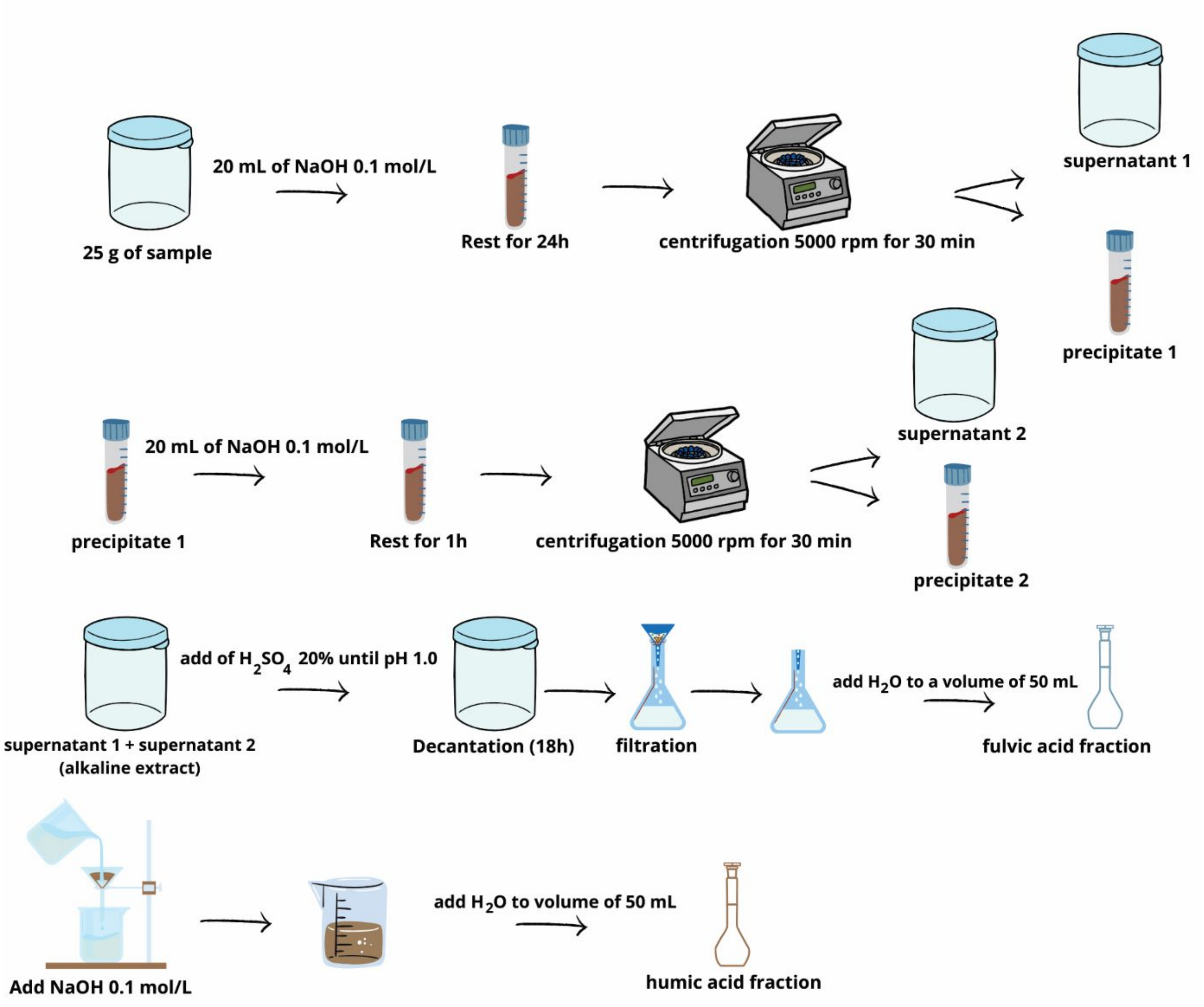

Figure S4. Methodology used to extract HA was adapted from Anielak *et al.*[19]

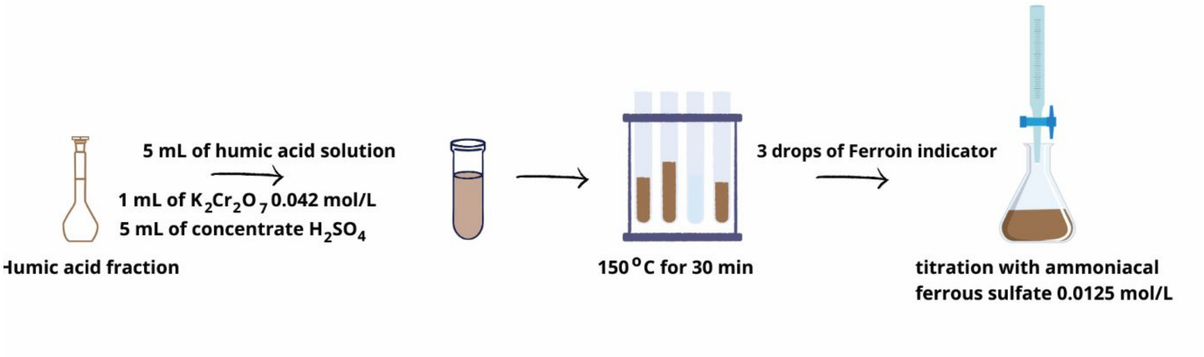

Figure S5. Methodology used to quantify HA adapted from Anielak *et al.*[19]

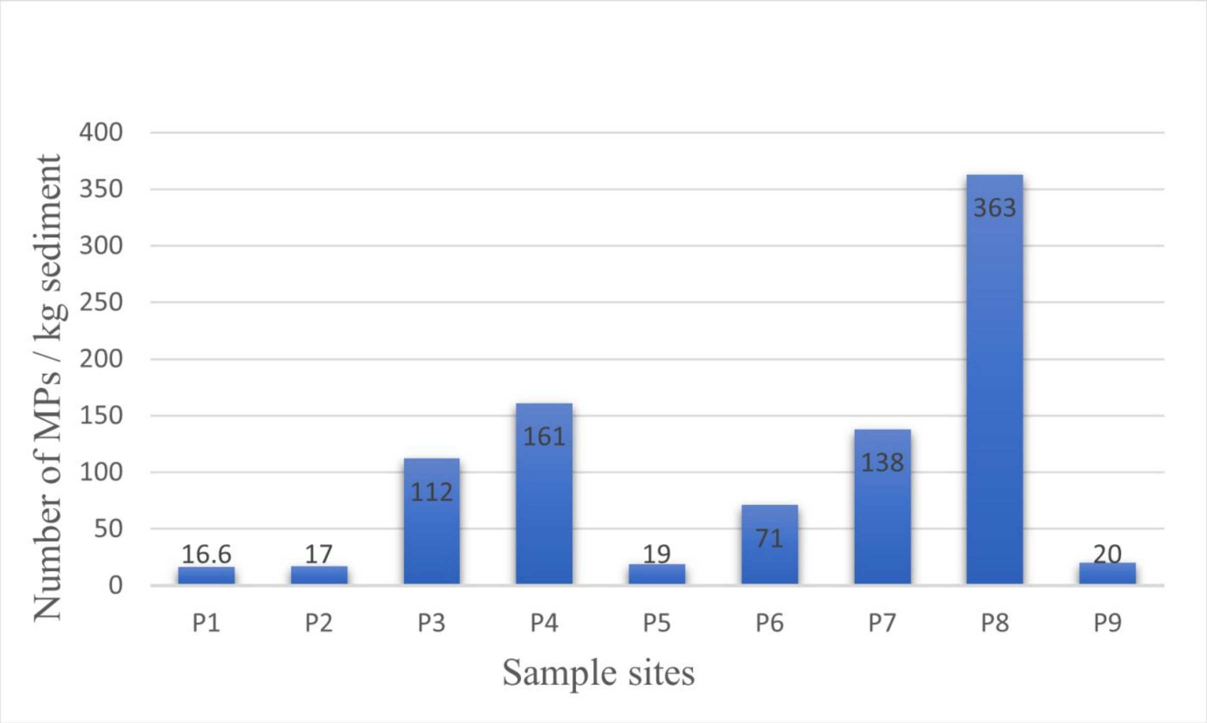

Figure S6. Number of microplastics per kilogram of sediment at sample sites.

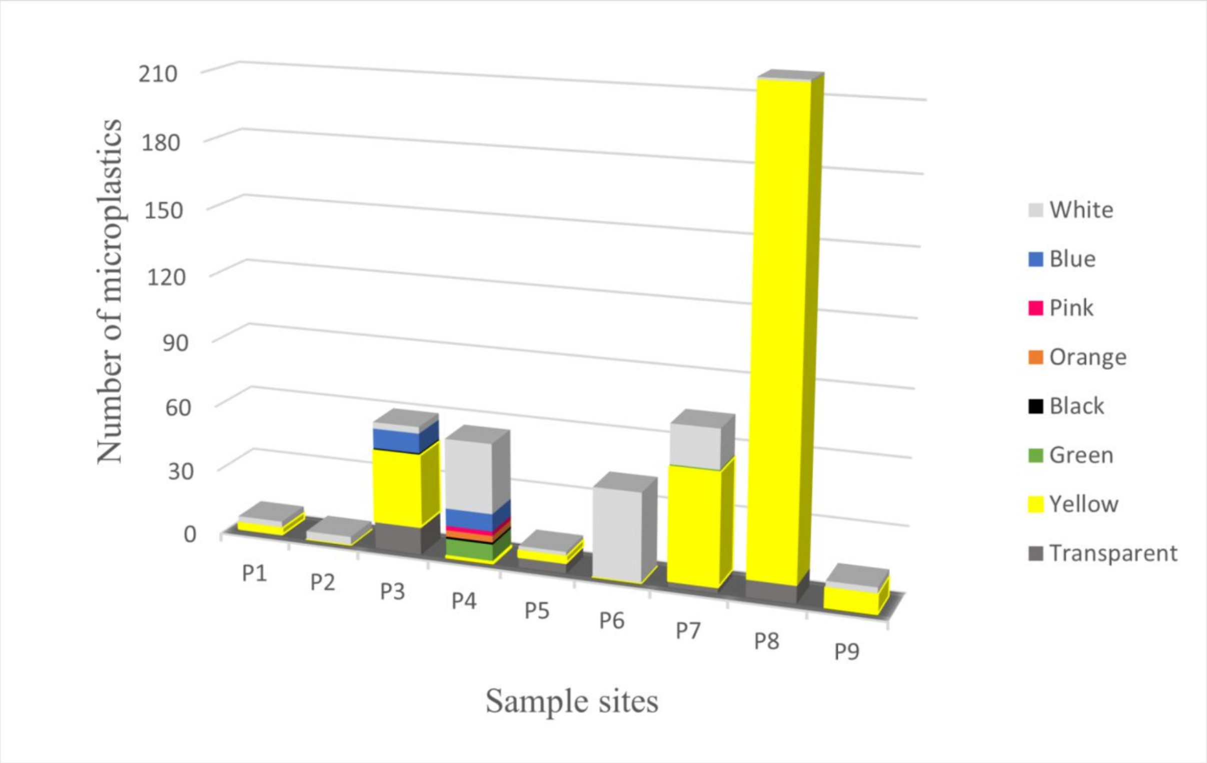

Figure S7. Number and color distribution of microplastics at different sample sites.

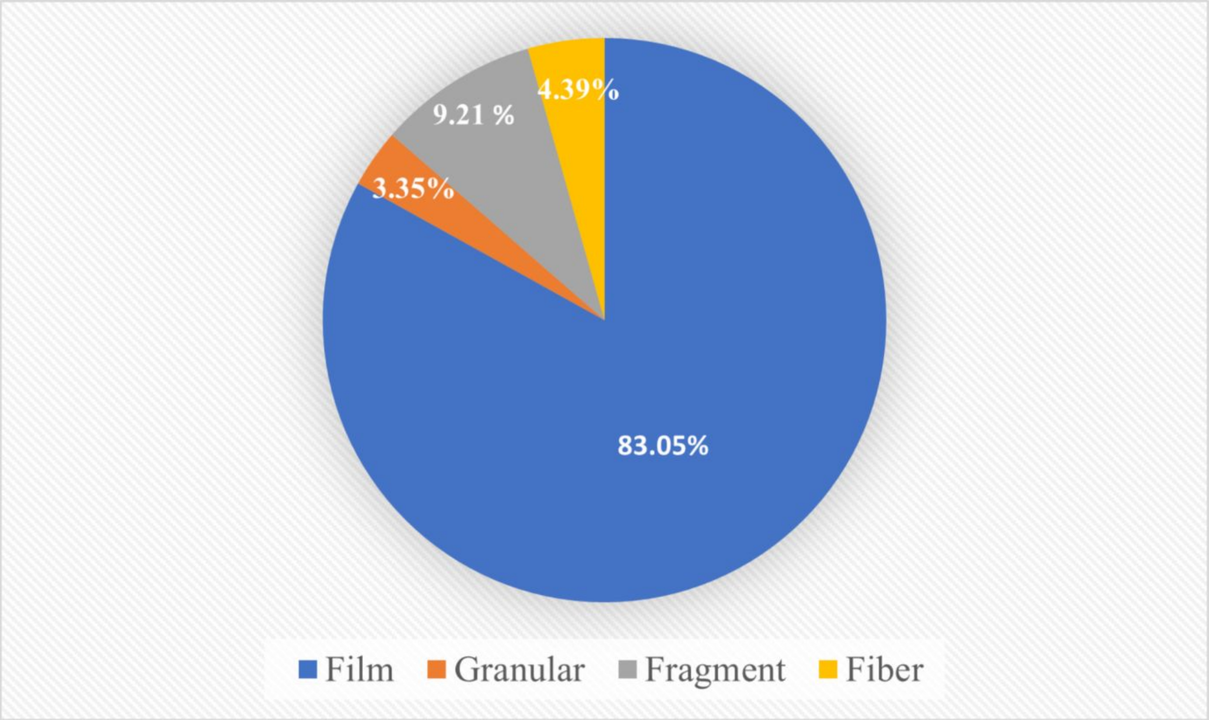

Figura S8. Percentage distribution of types of microplastics types.

References

(1) Thacharodi, A.; Meenatchi, R.; Hassan, S.; Hussain, N.; Bhat, M. A.; Arockiaraj, J.; Ngo, H. H.; Le, Q. H.; Pugazhendhi, A. Microplastics in the Environment: A Critical Overview on Its Fate, Toxicity, Implications, Management, and Bioremediation Strategies. *J. Environ. Manage.* **2023**, *349* (119433), 119433.

(2) Montagner, C.; Dias, M.; Paiva, E.; Vidal, C. MICROPLÁSTICOS: OCORRÊNCIA AMBIENTAL E DESAFIOS ANALÍTICOS. *Quim. Nova* **2021**. https://doi.org/10.21577/0100-4042.20170791.

(3) Shen, M.; Song, B.; Zeng, G.; Zhang, Y.; Huang, W.; Wen, X.; Tang, W. Are Biodegradable Plastics a Promising Solution to Solve the Global Plastic Pollution? *Environ. Pollut.* **2020**, *263* (Pt A), 114469.

(4) Allen, S.; Allen, D.; Phoenix, V. R.; Le Roux, G.; Durántez Jiménez, P.; Simonneau, A.; Binet, S.; Galop, D. Atmospheric Transport and Deposition of Microplastics in a Remote Mountain Catchment. *Nat. Geosci.* **2019**, *12* (5), 339–344.

(5) Vermeiren, P.; Ikejima, K.; Uchida, Y.; C Muñoz, C. Microplastic Distribution among Estuarine Sedimentary Habitats Utilized by Intertidal Crabs. *Sci. Total Environ.* **2023**, *866* (161400), 161400.

(6) Prata, J. C.; da Costa, J. P.; Duarte, A. C.; Rocha-Santos, T. Methods for Sampling and Detection of Microplastics in Water and Sediment: A Critical Review. *Trends Analyt. Chem.* **2019**, *110*, 150–159.

(7) Sunil, M.; Mithun; Kalthur, G.; Nair, M. P.; Gopinath, A.; Chidangil, S.; Kumar, S.; Lukose, J. Analysis of Microplastics in the Estuary Lying along the Coastal Belt of the Arabian Sea. *Case Stud. Chem. Environ. Eng.* **2024**, *10* (100804), 100804.

(8) Ivleva, N. P.; Wiesheu, A. C.; Niessner, R. Microplastic in Aquatic Ecosystems. *Angew. Chem. Int. Ed Engl.* **2017**, *56* (7), 1720–1739.

(9) De-la-Torre, G. E. Microplastics: An Emerging Threat to Food Security and Human Health. *J. Food Sci. Technol.* **2020**, *57* (5), 1601–1608.
(10) Taurozzi, D.; Scalici, M. Seabirds from the Poles: Microplastics Pollution Sentinels. *Front. Mar. Sci.* **2024**, *11*. https://doi.org/10.3389/fmars.2024.1343617.
(11) Bhuyan, M. S. Effects of Microplastics on Fish and in Human Health. *Front. Environ. Sci.* **2022**, *10* (827289). https://doi.org/10.3389/fenvs.2022.827289.
(12) Chen, Y.; Tang, H.; Cheng, Y.; Huang, T.; Xing, B. Interaction between Microplastics and Humic Acid and Its Effect on Their Properties as Revealed by Molecular Dynamics Simulations. *J. Hazard. Mater.* **2023**, *455* (131636), 131636.
(13) Chen, W.; Ouyang, Z.-Y.; Qian, C.; Yu, H.-Q. Induced Structural Changes of Humic Acid by Exposure of Polystyrene Microplastics: A Spectroscopic Insight. *Environ. Pollut.* **2018**, *233*, 1–7.
(14) Zhang, J.; Zhan, S.; Zhong, L.-B.; Wang, X.; Qiu, Z.; Zheng, Y.-M. Adsorption of Typical Natural Organic Matter on Microplastics in Aqueous Solution: Kinetics, Isotherm, Influence Factors and Mechanism. *J. Hazard. Mater.* **2023**, *443* (Pt A), 130130.
(15) Akkajit, P.; Tipmanee, D.; Cherdsukjai, P.; Suteerasak, T.; Thongnonghin, S. Occurrence and Distribution of Microplastics in Beach Sediments along Phuket Coastline. *Mar. Pollut. Bull.* **2021**, *169* (112496), 112496.
(16) Masura, J.; Baker, J. E.; Foster, G. D. (gregory D.; Arthur, C.; Herring, C. *Laboratory methods for the analysis of microplastics in the marine environment : recommendations for quantifying synthetic particles in waters and sediments*. https://repository.library.noaa.gov/view/noaa/10296 (accessed 2025-11-10).
(17) Harris, P. T. The Fate of Microplastic in Marine Sedimentary Environments: A Review and Synthesis. *Mar. Pollut. Bull.* **2020**, *158* (111398), 111398.
(18) Jha, P.; Biswas, A. K.; Lakaria, B. L.; Saha, R.; Singh, M.; Rao, A. S. Predicting Total Organic Carbon Content of Soils from Walkley and Black Analysis. *Commun. Soil Sci. Plant Anal.* **2014**, *45* (6), 713–725.
(19) Anielak, A. M.; Świderska-Dąbrowska, R.; Łomińska-Płatek, D.; Dąbrowski, T.; Piaskowski, K. Methods for Obtaining Humus Substances: Advantages and Disadvantages. *Appl. Sci. (Basel)* **2025**, *15* (5), 2463.
